# Stereological evaluation of the neuroprotective effects of curcumin on the spinal cord in a streptozotocin-induced diabetic rat model

**DOI:** 10.3389/fnana.2026.1822774

**Published:** 2026-05-01

**Authors:** Burcu Delibaş, Ayşe İkinci Keleş, Arife Ahsen Kaplan, Berk Kocaman, Gamze Altun, Kıymet Kübra Tüfekci, Abit Aktaş, Süleyman Kaplan

**Affiliations:** 1 Faculty of Medicine, Recep Tayyip Erdoğan University, Rize, Türkiye; 2 Faculty of Medicine, Aksaray University, Aksaray, Türkiye; 3 Faculty of Medicine, Bolu Abant Izzet Baysal University, Bolu, Türkiye; 4 Faculty of Veterinary Medicine, Istanbul Universitesi-Cerrahpasa, Avcılar, Türkiye; 5 Faculty of Medicine, Ondokuz Mayis University, Samsun, Türkiye; 6 Department of Histology and Embryology, Faculty of Medicine, Kastamonu University, Kastamonu, Türkiye

**Keywords:** cervical segment, curcumin, diabetes mellitus, disector, spinal cord

## Abstract

**Introduction:**

This study examined how curcumin influences spinal cord morphological parameters in rats with STZ-induced diabetes using unbiased stereological methods.

**Methods:**

Fifty-six female Wistar albino rats were randomly divided into seven experimental groups (*n* = 8): Control, Sham, Curcumin, Diabetes Mellitus (DM), DM + Curcumin after 7 days (DC1), DM + Curcumin after 21 days (DC2), and DM + Curcumin simultaneously (DC3). Diabetes was induced via a single intraperitoneal dose of STZ (50 mg/kg). Curcumin was administered at a dose of 30 mg/kg via intragastric gavage for 14 consecutive days. C3–C5 spinal segments were collected at the end of the experiment, processed for histology, and stained with toluidine blue and cresyl violet for stereological analysis. Neuronal quantification in the anterior horn was performed using physical fractionator. The volume fractions of the spinal cord, including white matter (WM/total volume) and gray matter (GM/total volume), were estimated using the Cavalieri’s principle.

**Results:**

The diabetic (DM) group showed a significant reduction in motor neuron number compared with the Control group (*p* = 0.019), demonstrating diabetes-induced neuronal loss. In contrast, the DC2 treatment group showed a significant increase in motor neuron counts compared with DM (*p* = 0.04), suggesting a possible neuroprotective effect of curcumin. Total spinal cord volume did not differ significantly among groups. WM/Total ratio decreased in the Sham group but increased with curcumin (DC3). GM/Total ratio was lower in DC3 than Sham, and curcumin produced a non-significant improvement compared with diabetic rats. Increased caspase-3 immunoreactivity in the diabetic group indicates activation of apoptotic pathways, consistent with the observed reduction in motor neuron number and soma size. Furthermore, the marked increase in GFAP immunoreactivity, particularly in the DC2 group, reflects astrocyte activation and a reactive gliosis, which are commonly associated with metabolic stress and neuroinflammation in diabetic conditions.

**Discussion:**

Curcumin administration partially mitigated spinal motor neuron loss induced by experimental diabetes. The timing of curcumin treatment influenced its efficacy. These findings suggest that curcumin may have therapeutic potential for preventing diabetes-induced spinal cord neurodegeneration.

## 1 Introduction

Neurodegeneration in diabetes mellitus is unique, characterized by terminal retraction, a process in which nerve terminals are lost while the perikarya, or cell bodies, remain intact. Sensory neurons undergo more severe impairment, characterized by diminished conduction velocity, perikaryal and axonal atrophy, suppressed synthesis of structural proteins, and degeneration of terminal epidermal axons (Zochodne and Ho, 1992; Kennedy and Zochodne, 2000, 2005). For instance, streptozotocin-induced Type I diabetic mice exhibit distal sural axonal degeneration, atrophy and loss of sensory neuron perikarya, and depletion of epidermal axons (Kennedy and Zochodne, 2000). The vulnerability of motor neurons might lie in their anatomical position. Motor neuron perikarya are sheltered within the spinal cord, whereas sensory neurons reside in the dorsal root ganglia, which have weaker blood–tissue barrier protection compared to the spinal cord (Arvidson, 1979).

Curcumin with the chemical formula [1,7-bis(4-hydroxy-3-methoxyphenyl)-1,6-heptadiene-3,5-dione] is a naturally occurring, nonsteroidal compound. It possesses a broad spectrum of pharmacological properties, including anti-inflammatory, anticarcinogenic, antimicrobial, antioxidant, and hypocholesterolemic activities (Shishodia et al., 2005; Chen et al., 2006). Additionally, curcumin has been shown to counteract ultraviolet (UV) radiation–induced apoptotic alterations, including mitochondrial membrane potential dissipation, cytochrome c release, and increased reactive oxygen species (ROS) production. Recent animal studies indicate that curcumin exerts notable neuroprotective effects in both diabetic conditions and spinal cord pathologies by modulating oxidative stress, suppressing neuroinflammatory responses, and regulating glial cell activation. For example, in a rat model of diabetes induced by streptozotocin (STZ), curcumin reduced mechanical allodynia/hyperalgesia and lowered the levels of TNF-*α* and TNF-α receptor 1 in the dorsal horn of the spinal cord (Li et al., 2013). In a more recent study of diabetic central neuropathy, curcumin attenuated neuronal apoptosis and glial activation in the spinal cord by activating the Nrf2/HO-1 pathway and inhibiting NF-κB signaling (Elsayed et al., 2023). Together, these data suggest that curcumin may potentially benefit diabetic spinal cord complications by reducing oxidative damage, inhibiting pro-inflammatory signaling and improving neuronal/glial survival.

The aim of this study was to investigate changes in the total number of neurons within the gray matter (GM) of the cervical spinal cord in rats with experimentally induced diabetes, using unbiased stereological methods. Alongside estimating motor neuron numbers, we evaluated spinal cord volume and the volume fractions of GM and white matter (WM) in both healthy and diabetic animals. The C3–C5 cervical spinal cord region was specifically chosen for assessment of potential pathological alterations, as this segment is commonly affected in various traumatic and degenerative conditions in humans. This spinal cord segment is particularly relevant in diabetes because it contributes to the neural pathways governing autonomic regulation, respiratory control, and metabolic homeostasis (Maser et al., 2003; Mantilla et al., 2018). Consequently, we anticipated that establishing normative morphometric data for the rat cervical spinal cord would provide valuable insights into studies of spinal cord disorders and diverse experimental models of diabetes.

## 2 Materials and methods

### 2.1 Animals and experimental groups

All experimental procedures were conducted in accordance with the approved guidelines of the Ondokuz Mayıs University Animal Experiments Local Ethics Committee (approval no: 2017/53), and permission was received from the Ondokuz Mayıs University Animal Experiments Local Ethics Committee (Petition no: E-68489742-604.02.2500247693, date: 30.10.2025). All surgical and experimental procedures were performed in accordance with ARRIVE and the Guide for the Care and Use of Laboratory Animals. Adult female Wistar albino rats weighing between 250 and 300 g were used in the study. The animals were randomly assigned to seven groups (*n* = 8 per group). They were housed in stainless steel cages under controlled conditions, including a 12-h light/12-h dark cycle, a constant temperature of 22 ± 2 °C, and a relative humidity of 50–60%. Rats had ad libitum access to standard pellet feed and water. A detailed description of the experimental groups is provided in Table 1.

**Table 1:** All study groups are shown in the following table (*n* = 8).

Control group (Cont)	Healthy animals that received no treatment, serving as the baseline reference
Sham group (Sham)	Healthy animals that received corn oil (vehicle for curcumin) via oral gavage once daily for 14 days.
Curcumin (Cur)	Healthy animals treated with 30 mg/kg curcumin dissolved in corn oil, administered orally for 14 consecutive days.
Diabetes mellitus (DM)	Diabetes was induced by a single STZ injection. No treatment was given after confirmation of hyperglycemia.
DM + Curcumin from day 7 (DC1)	Curcumin treatment (30 mg/kg) started on day 7 after STZ injection and continued for 14 days.
DM + Curcumin from day 21 (DC2)	Curcumin treatment (30 mg/kg) started on day 21 post-STZ and continued for 14 days.
DM + Curcumin simultaneously (DC3)	Curcumin (30 mg/kg) was administered simultaneously with STZ and continued daily for 14 days.

### 2.2 Induction of diabetes

After overnight fasting, diabetes was induced by intraperitoneal injection of STZ (50 mg/kg) dissolved in 0.1 M citrate buffer (pH 4.0). To prevent acute hypoglycemia, 5% glucose was supplied in drinking water for 24 h post-injection. At 72 h following STZ administration, fasting blood glucose levels were measured to confirm the induction of diabetes. The rats were fasted overnight, and blood samples were collected from the tail vein. Glucose levels were determined by applying a drop of blood onto a glucometer test strip (Gl 300 Plus MED). For each animal, three measurements were obtained, and rats with fasting blood glucose levels of ≥250 mg/dL were considered as diabetic and included in the study (Wu and Huan, 2008). Animals in the Control, Sham, and Curcumin-only groups also received a single intraperitoneal injection of 0.1 M citrate buffer (pH 4.0), which served as the vehicle for STZ, to ensure consistency across experimental conditions. During the experimental period, four rats died—two in the DM group and two in the DC1 group. Furthermore, two rats failed to develop hyperglycemia after STZ injection. These animals were replaced with new animals that underwent identical experimental procedures to maintain *n* = 8 per group for statistical analysis. This replacement protocol was implemented to ensure adequate statistical power while maintaining ethical standards. The timing of replacement and the consistency of experimental conditions were carefully controlled to minimize potential bias. All animals, including replacements, were subjected to the same housing conditions, diabetes induction protocol, and treatment regimen.

### 2.3 Tissue collection and histological preparation

Upon completion of the experiment, the animals were euthanized, and spinal cord segments from the C3–C5 region were carefully harvested. The tissues were fixed and embedded in resin according to standard protocols. Consecutive semi-thin sections (1 μm thick) were obtained using an ultramicrotome and stained with toluidine blue for histological and stereological analysis.

### 2.4 Stereological analysis

Cervical spinal cord sections were stained with 0.1% cresyl violet and evaluated according to a standard protocol for assessing motor neuron integrity based on Nissl substance. The number of motor neurons in the ventral horn of the cervical spinal cord was estimated using the physical fractionator, an unbiased stereological cell-counting technique. Analyses were performed with a Nikon Eclipse 600 microscope and ImageJ software.

A virtual grid (150 × 150 μm) and a counting frame (75 × 75 μm) were applied, optimized to ensure counting of at least 200 cells per animal with error coefficients <0.07. Anatomical structures were outlined using a 10×/0.45 objective, while motor neuron quantification was conducted with a 40×/1.40 objective. Motor neurons located in lamina IX (diameter >30 μm) with a prominent nucleus and an average orthogonal diameter were counted at discrete levels of the cervical spinal cord (C3–C5), with 15 sections per level per spinal cord segment per group, separated by approximately 120 μm. The results were expressed as averages per ventral horn, considering both sides of the spinal cord. In addition to stereological counting, motor neuron morphologies were analyzed in the cervical spinal cord regions. Motor neurons in the anterior horn were quantified using the physical fractionator with ImageJ software and an unbiased counting frame. The analysis followed Gundersen’s principles for unbiased stereology.

The volume of each spinal cord region was estimated using Cavalieri’s principle, as described by Gundersen and Jensen (1987). To apply this method, a randomly positioned uniform point-counting grid was superimposed on the section images. The volumes of the total spinal cord, gray matter (GM), white matter (WM), and central canal (CC) were then calculated using the following formula:


Volume=(a/p)×ΣP×t×ssf

In this formula, *a*/*p* represents the area associated with each point on the point-counting grid, *ΣP* is the total number of points intersecting the section surface, *t* denotes the thickness of the sections, and *ssf* refers to the section sampling fraction.

Each section analyzed along the horizontal axis and MNs belonging to medial and lateral columns of the ventral horn are counted with physical fractionator at 40× magnification. Within counting frames of 40 μm × 40 μm MNs with a cell body diameter >30 μm are counted at 100× magnification. The total number of MNs are calculated by using the following formula: N=Total number(Q)×(1/ssf)×(1/asf) (*Q* = refers to the total number of cells counted in each dissector; *ssf* (*s*ection *s*ampling *f*raction) is the ratio of the number of regularly spaced sections used for counting to the total number of sections across the cervical spinal cord. In the present calculations, this corresponds to 1/5, since one out of every 5 slices (40 of 200) was analyzed; *asf* (*a*rea *s*ampling *f*raction) is calculated as the disector area divided by the reference area between disectors. In this case, it corresponds to 1,600 μm^2^ × the number of disectors/total regional area.

### 2.5 Immunohistochemical analysis

One cervical spinal cord sample (C3–C5 segment) from each experimental group was fixed in 4% paraformaldehyde solution for 48 h at 4 °C. Following routine histological processing, the tissues were embedded in paraffin, and 5-μm-thick serial sections were obtained from each block.

Immunohistochemical staining procedures were performed on these sections to evaluate apoptotic activity and astroglial activation. Anti-caspase-3 (dilution 1:200; ab4051, Abcam, RRID: AB 304243) was used as a marker of apoptosis, while anti-GFAP (dilution 1:200, MAB360, Sigma, RRID: AB_11212597) was used to assess astrocyte activation. After deparaffinization and rehydration, antigen retrieval was performed in citrate buffer (pH 6.0) using a microwave heating method. Endogenous peroxidase activity was blocked with 3% hydrogen peroxide for 10 min. Non-specific binding was prevented by incubation with normal serum for 20 min at room temperature. The sections were then incubated with primary antibodies overnight at 4 °C. After washing in phosphate-buffered saline (PBS), sections were incubated with appropriate biotinylated secondary antibodies and subsequently treated with an HRP/AEC (ABC) detection kit (Abcam, UK). Immunoreactivity was visualized using 3,3′-diaminobenzidine (DAB) as the chromogen. Finally, sections were counterstained with hematoxylin, dehydrated, and coverslipped. Negative control sections were processed identically, except that the primary antibody was omitted. No quantitative or semi-quantitative measurements were performed; instead, immunohistochemical findings were assessed solely on morphological and observational criteria, including distribution, localization, and staining intensity.

### 2.6 Statistics

Statistical analyses were performed using GraphPad Prism 8 (GraphPad Software, La Jolla, CA, USA). A *p*-value < 0.05 was considered statistically significant. Data normality was assessed prior to analysis. For normally distributed data, one-way ANOVA followed by Tukey’s *post hoc* test was applied.

## 3 Results

### 3.1 Neuronal cell counting

**Figure 1:**
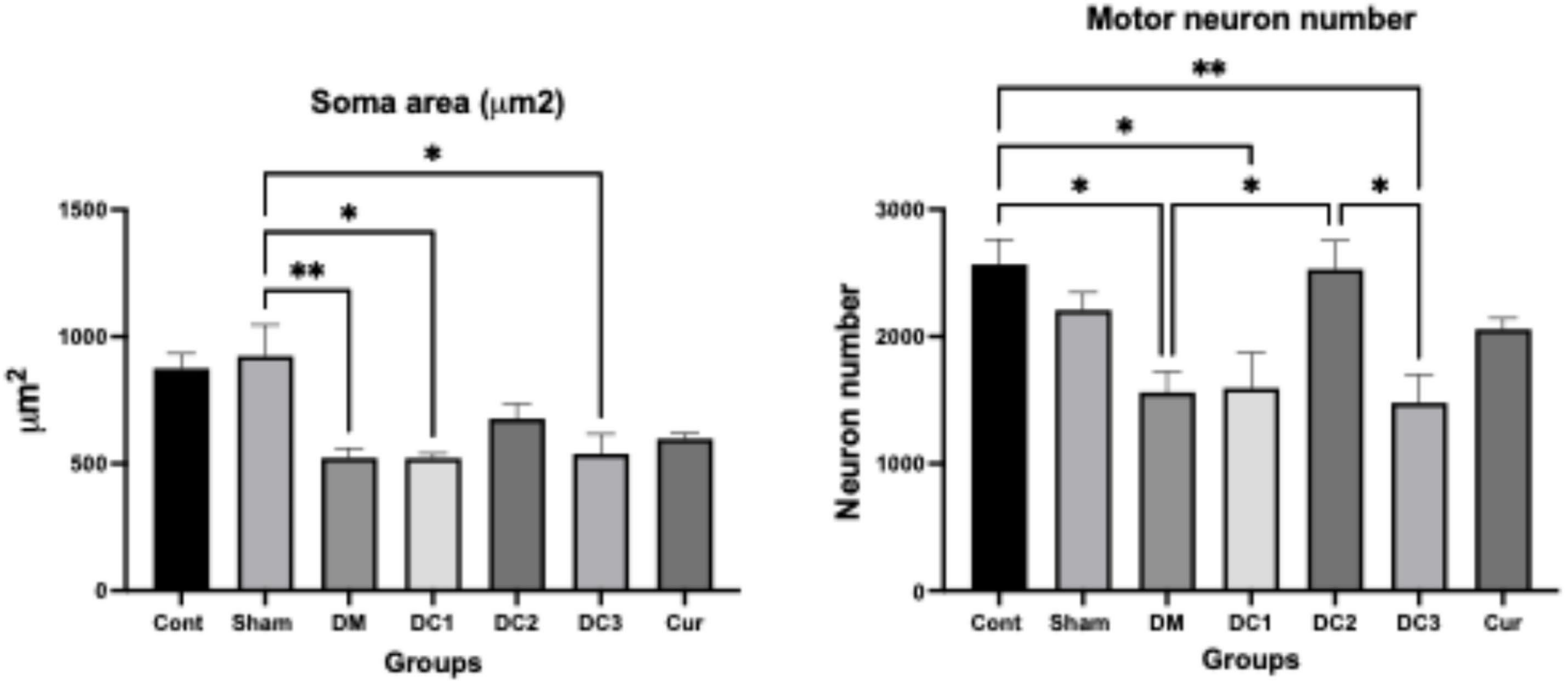
Differences in motor neuron soma area among the seven experimental groups (Cont, Sham, DM, DC1, DC2, DC3, Cur) were evaluated using one-way ANOVA, followed by Tukey’s *post hoc* test to identify pairwise differences. *Post hoc* comparisons indicated that the DM group showed a significant reduction in soma area compared with the control group (*p* = 0.007). Curcumin treatment groups (DC1, DC2, DC3, Cur) demonstrated partial restoration of soma size compared with the DM group, but the results were not found statistically significant (*p* > 0.05). Significant differences were found between the Sham and DM, Sham and DC1, and Sham and DC3 groups, with *p*-values of 0.007, 0.03, and 0.016, respectively. Regarding motor neuron numbers, the DM group showed a significant decrease compared with the control (*p* = 0.019). DC2 showed a significant increase in neuron number compared with DM (*p* = 0.04), suggesting a possible neuroprotective effect. Data are expressed as mean ± SD (*p ≤ 0.05, **p ≤ 0.01).

Estimated motor neuron number and soma value areas are shown in the following table and graph (Table 2; Figure 1).

**Table 2:** Estimated counts of total motor neurons, and soma areas (μm^2^), along with the coefficient of error (CE) and coefficient of variance (CV) from stereological analysis of the cervical spinal cord in all groups.

Parameter	Control	Sham	DM	DC1	DC2	DC3	Cur.
Estimated motor neuron number	2,568 ± 394	2,208 ± 311	1,561 ± 324	1,593 ± 483	2,529 ± 388	1,479 ± 492	2058 ± 147
CE	0.08	0.05	0.06	0.06	0.09	0.08	0.05
CV	15.34	14.09	20.76	30.32	15.34	33.27	7.14
vs Control (*p*)	–	0.79	0.019*	0.044*	>0.99	0.006**	0.6
vs DM (*p*)	0.019*	0.20	–	–	0.046*	0.99	0.62
Soma area	875 ± 114	924 ± 298	523 ± 90	521 ± 31	677 ± 99	532 ± 173	598 ± 37
CE	0.06	0.07	0.08	0.05	0.1	0.09	0.1
CV	13.03	32.25	17.21	5.95	14.62	32.51	6.19
vs Control (*p*)	–	0.99	**0.053**	0.14	0.73	0.09	0.37
vs Sham (*p*)	0.99	–	0.007**	0.03*	0.41	0.016*	0.14
vs DM (*p*)	**0.053**	0.007**	–	>0.99	0.85	>0.99	0.99

The values were represented as mean ± SD. Statistical comparisons were performed using one-way ANOVA followed by *post-hoc* tests. The *p*-values of statistically significant variables were shown in bold with asterix (*p ≤ 0.05, **p ≤ 0.01).

### 3.2 Volume fractions of white and gray matter of the spinal cord

The volume fraction of a given component relative to its reference volume represents a fundamental metric extensively used in biomedical investigations (Gundersen, 1986). It represents the proportion of a specific phase or component relative to the entire structure. The volume fraction of phase X within a reference volume Y can be calculated using the following expression:


$$V_{v}\;(X,Y)=\frac{\text{Volume of}\;X\;\text{phase in}\;Y\;\text{reference space}}{\text{Volume of}\;Y\;\text{reference space}}$$

**Figure 2:**
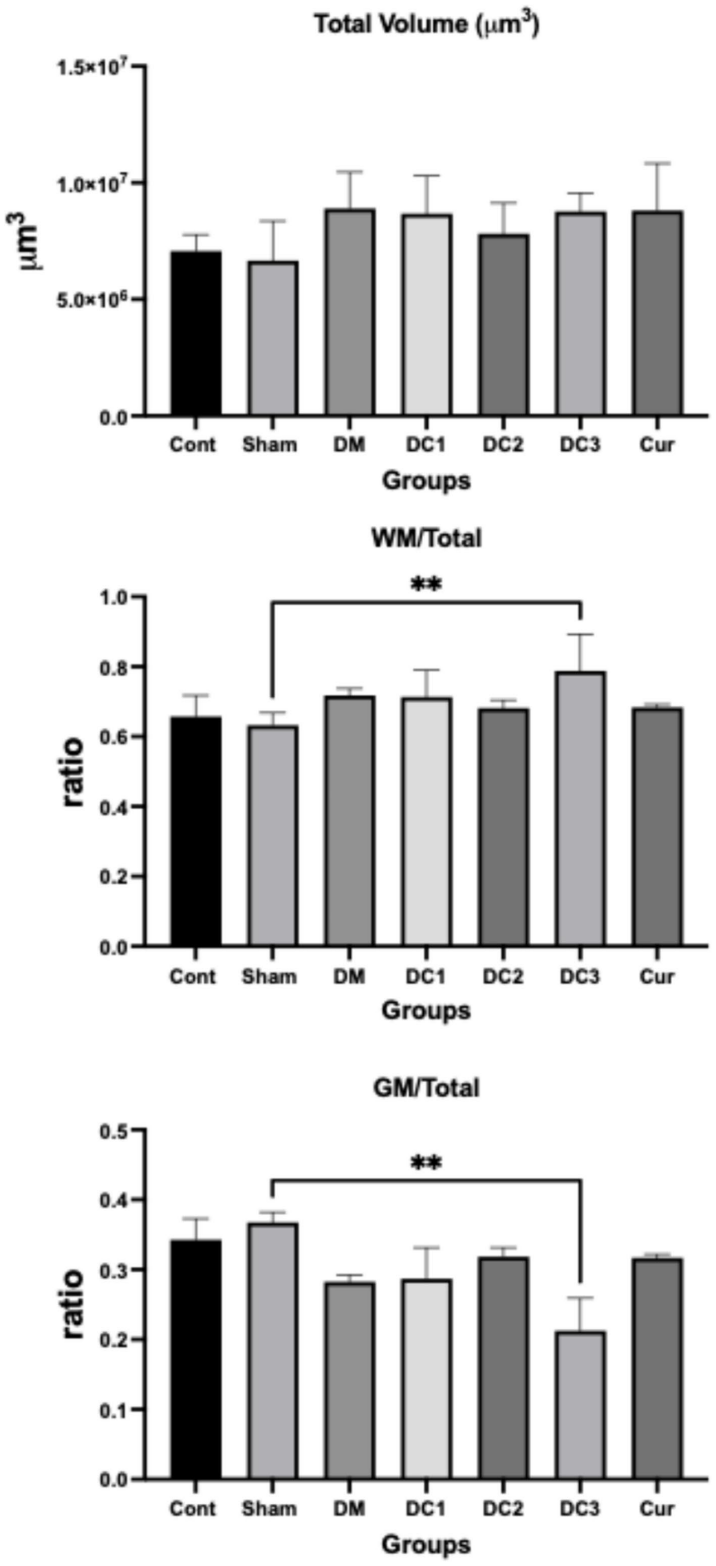
To compare total spinal cord volume among experimental groups (Cont, Sham, DM, DC1, DC2, DC3, Cur), a one-way analysis of variance (ANOVA) was performed. The analysis showed no statistically significant difference in total volume between groups (*p* > 0.05). Although the diabetic (DM) and curcumin-treated (Cur) groups exhibited slightly higher mean values than the control group, these differences did not reach statistical significance.

Here, V_V_ (X, Y) denotes the volume fraction of phase X within the reference volume Y. For example, using this method, the volume fraction of gray matter within the spinal cord—V_V_ (gray matter, spinal cord)—can be estimated. The volume fraction ranges from 0 to 1 and is typically presented as a percentage (Gundersen and Jensen, 1987). Based on the information above, the same point counts used for total volume estimation were also applied to calculate the volume fraction of gray or white matter within the spinal cord segment. Estimated values of total volume, WM/Total volume, and GM/Total volume are shown in the following Table 3 and Figure 2.

**Table 3:** Estimated values of total volume (mm^3^), WM/Total volume, and GM/Total volume, along with the coefficient of error (CE) and coefficient of variance (CV) from stereological analysis of the cervical spinal cord in all groups.

Parameter	Control	Sham	DM	DC1	DC2	DC3	Cur.
Total volume	0.70 ± 0.07	0.66 ± 0.17	0.88 ± 0.15	0.86 ± 0.16	0.78 ± 0.13	0.87 ± 0.07	0.88 ± 0.20
CE	0.04	0.06	0.07	0.04	0.09	0.1	0.08
CV	10.00	25.76	17.05	18.60	16.67	8.05	22.73
WM/Total	0.65 ± 0.06	0.63 ± 0.03	0.71 ± 0.02	0.71 ± 0.07	0.68 ± 0.02	0.78 ± 0.10	0.68 ± 0.008
CE	0.08	0.1	0.09	0.08	0.05	0.06	0.1
CV	9.23	4.76	2.82	9.86	2.94	12.82	1.18
vs Sham (*p*)	0.99	–	0.33	0.51	0.9	0.0053**	0.88
GM/Total	0.34 ± 0.06	0.36 ± 0.03	0.28 ± 0.02	0.28 ± 0.07	0.31 ± 0.02	0.21 ± 0.10	0.31 ± 0.008
CE	0.07	0.06	0.1	0.1	0.9	0.1	0.8
CV	17.65	8.33	7.14	25.00	6.45	47.62	2.58
vs Sham (*p*)	0.99	–	0.33	0.51	0.9	0.0053**	0.88

The values were represented as mean ± SD. Statistical comparisons were performed using one-way ANOVA followed by *post-hoc* tests. The *p*-values of statistically significant variables were shown in bold with Asterix (*p ≤ 0.05, **p ≤ 0.01).

### 3.3 Light microscopic evaluation

**Figure 3:**
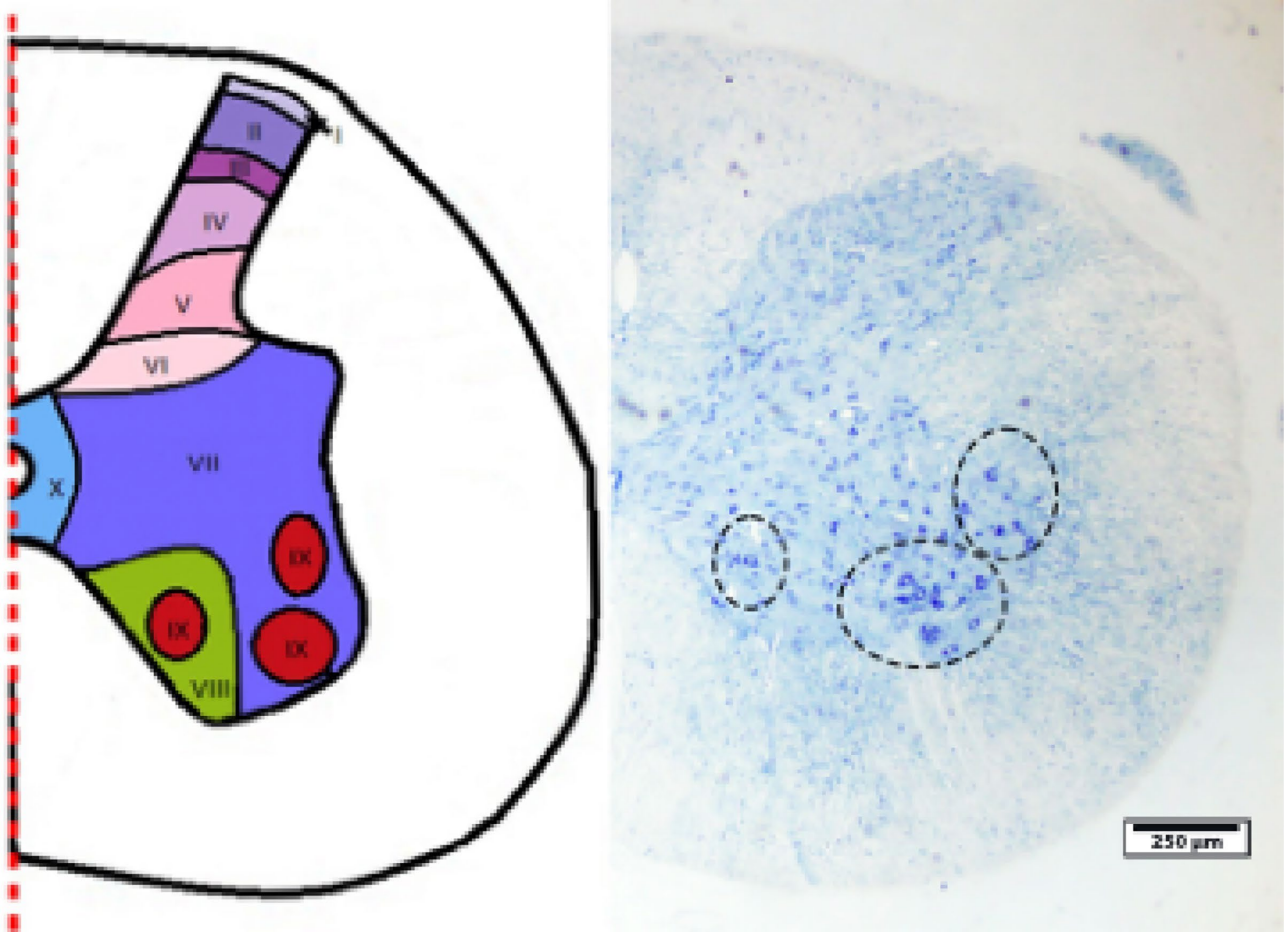
Schematic representation and histological localization of Rexed lamina IX in the spinal cord. The left panel illustrates the Rexed laminar organization of the spinal cord, with lamina IX highlighted in the ventral horn. The right panel shows a representative transverse section of the spinal cord used for stereological analysis. The region corresponding to lamina IX is delineated by dashed lines, indicating the anatomical boundaries within which all stereological measurements were performed. Scale bar = 250 μm.

**Figure 4:**
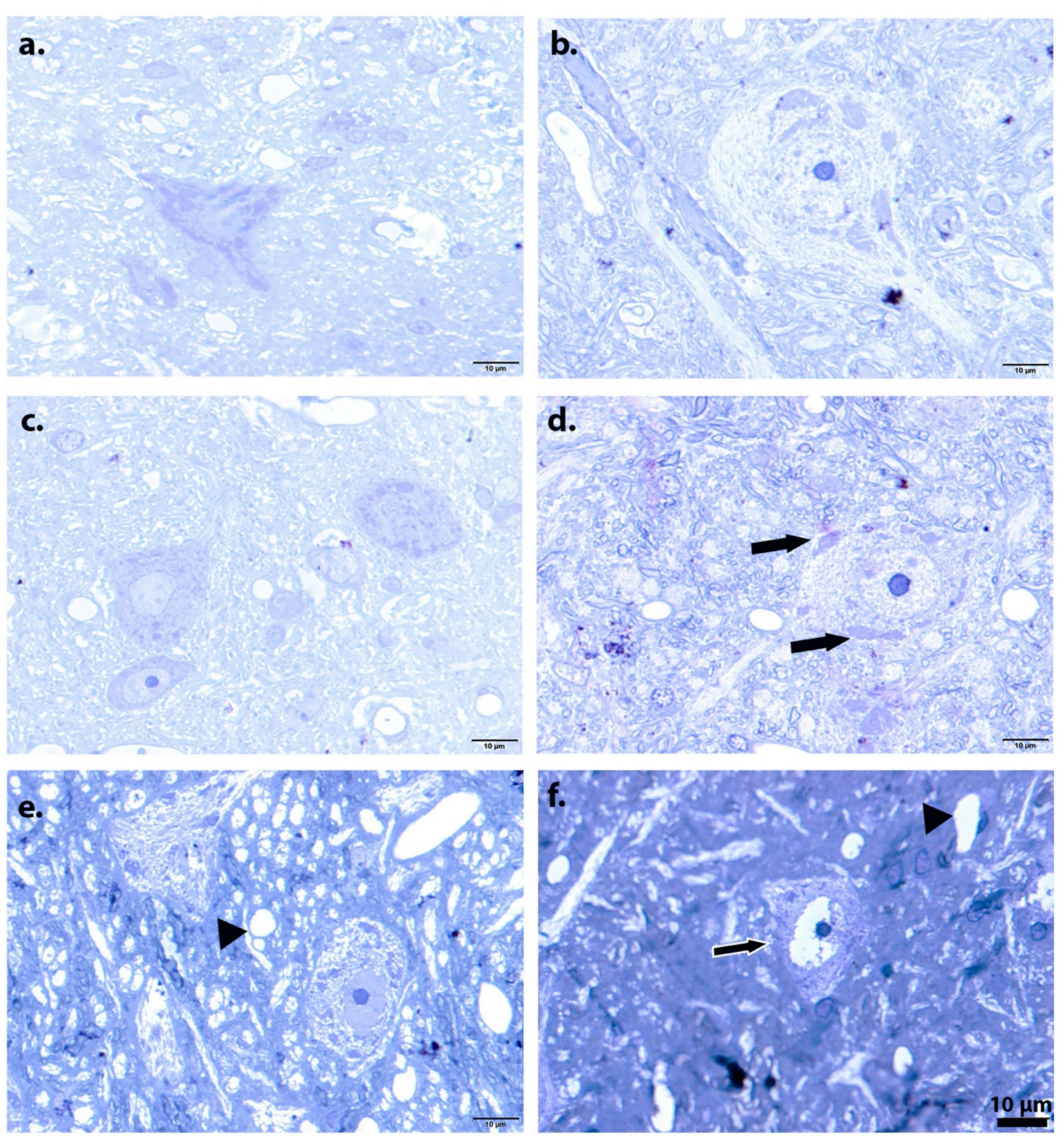
Motor neuron images obtained from groups, respectively, [**(a)** control; **(b)** sham, **(c)** curcumin, **(d)** DM, **(e)** DC1, **(f)** DC2]. **(a)** Control group showing a well-preserved motor neuron with normal soma contour, centrally located nucleus, and intact Nissl substance. **(b,c)** The sham group shows a typical motor neuron morphology comparable to that of the control. **(d)** The diabetic group exhibited reduced neuronal soma size, pale cytoplasm, and signs of chromatolysis with decreased Nissl substance (arrow). **(e)** Diabetic + treatment group (DC1) showing partial preservation of neuronal morphology, with a more distinct nucleus and improved cytoplasmic organization compared to the diabetic group. **(f)** Diabetic + treatment group (DC2) displaying pronounced neuropil vacuolization (arrowhead), increased extracellular spaces, and neuronal shrinkage. Severe pathological appearance characterized by marked cytoplasmic condensation (arrow with white contour), darkly stained nuclei, and extensive tissue disorganization (Toluidine blue staining bar: 10 μm).

Stereological and light microscopic evaluations were performed in Rexed lamina IX of the spinal cord. Lamina IX is in the ventral horn and contains the cell bodies of somatic motor neurons; therefore, it was selected as the target region for quantitative motor neuron analysis in this study (Figure 3). The anatomical localization of lamina IX within the spinal cord sections was determined according to the Rexed laminar organization. The boundaries of lamina IX corresponding to the evaluated regions are illustrated with dashed lines in the figure. These dashed outlines represent the approximate anatomical limits of lamina IX within the spinal cord cross-section and ensured that all stereological measurements were restricted exclusively to this defined region. All analyses were conducted in accordance with the principles of systematic, uniform random sampling. Toluidine blue–stained semi-thin sections illustrate morphological alterations in motor neurons and the surrounding neuropil (Figure 4).

Group comparisons for the white matter–to–total volume ratio (WM/Total) were analyzed using one-way ANOVA followed by Tukey’s *post hoc* test. A significant main effect of group was detected (*p* < 0.05). *Post hoc* analysis revealed that the Sham group showed a significantly lower WM/Total ratio than the Control group (*p* < 0.01). Curcumin treatment (DC3) increased the WM/Total ratio compared with the Sham group, (*p* = 0.0053).

The gray matter–to–total volume ratio (GM/Total) was similarly analyzed using one-way ANOVA with Tukey’s post hoc comparisons. A statistically significant difference was observed between the Sham and DC3 groups (*p* < 0.01). Post hoc testing indicated that the DC3 group had a significantly lower GM/Total ratio than the Sham group (*p* = 0.0053). Curcumin administration increased GM/Total compared with DM, but the difference was not statistically significant (*p* > 0.01). Data are presented as mean ± SD (*p ≤ 0.05, **p ≤ 0.01).

### 3.4 Immunohistochemical evaluation

**Figure 5:**
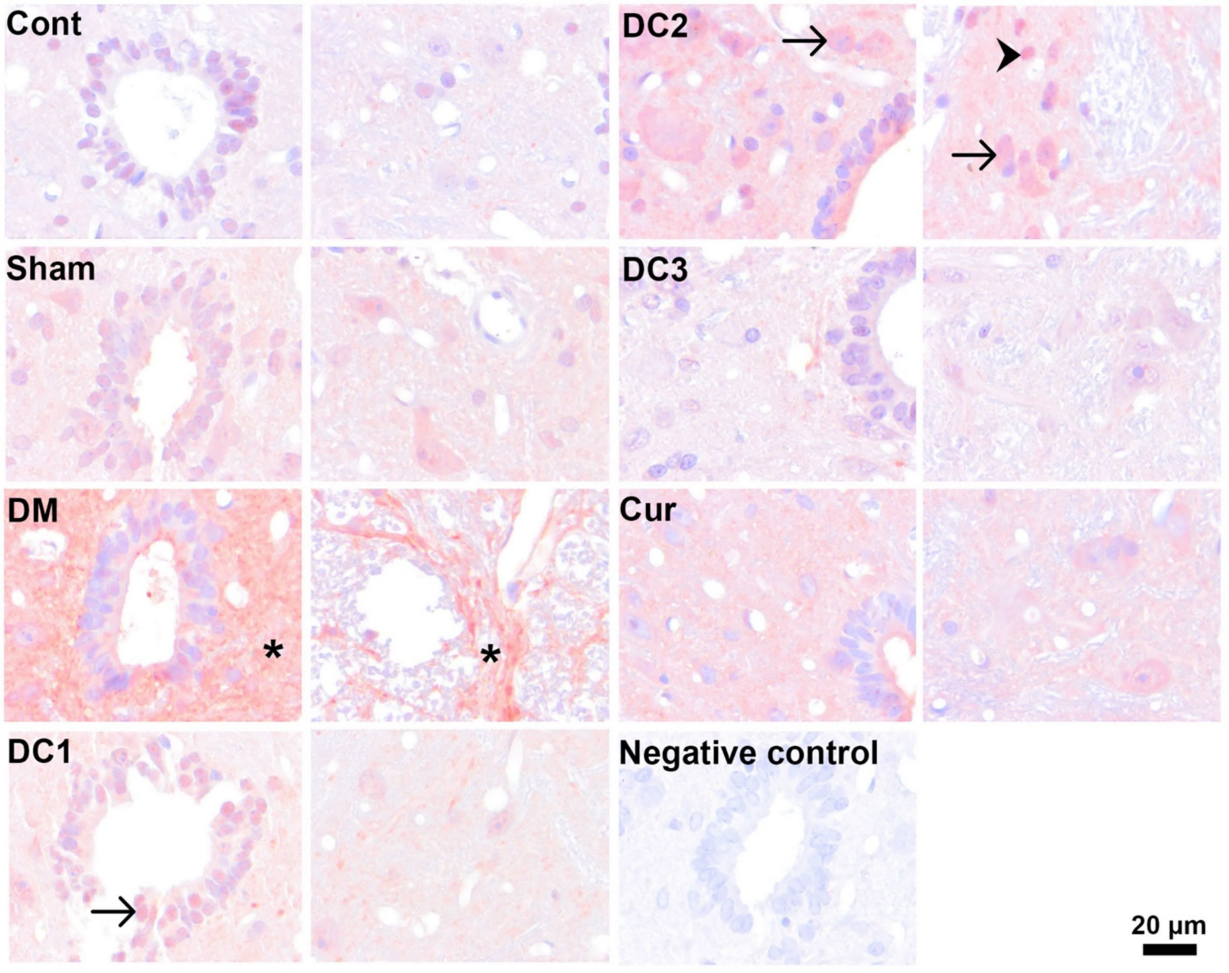
Representative immunohistochemical staining for caspase-3 in spinal cord tissue across experimental groups. Caspase-3 immunoreactivity is increased in both the DM and DC2 groups. In the DM group, widespread cytoplasmic caspase-3 immunoreactivity (*) is observed. In contrast, the DC2 group exhibits strong caspase-3 immunoreactivity in both neuronal cell bodies (arrows) and glial cells (arrowheads), indicating enhanced apoptotic activity across multiple cell populations. In the DC1 group, intense caspase-3 positivity is predominantly detected in ependymal cells. Scale bar = 20 μm.

**Figure 6:**
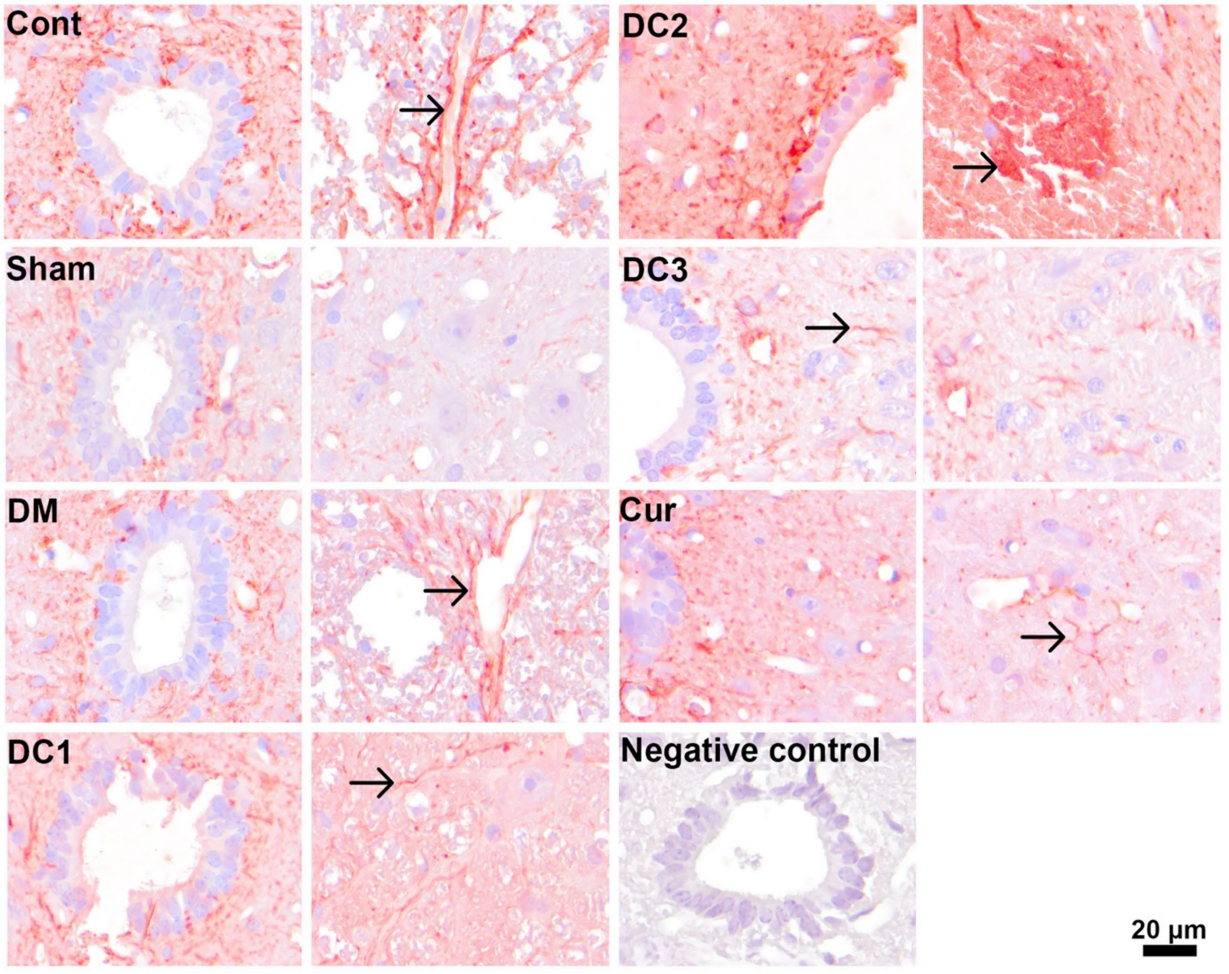
Representative immunohistochemical staining of glial fibrillary acidic protein (GFAP) in spinal cord tissue across experimental groups. GFAP immunoreactivity (red) is predominantly localized to astrocytic processes, while cell nuclei are counterstained with hematoxylin. In all groups, GFAP expression is mainly observed in astrocytic processes and is minimal within astrocyte cell bodies. A marked increase in GFAP immunoreactivity, indicative of enhanced astrocyte activation, is evident in the DC2 group compared with the other groups. Arrows indicate GFAP-positive astrocytic processes. Scale bar = 20 μm.

To further elucidate the cellular mechanisms underlying the stereologically observed neuronal alterations, immunohistochemical analyses were performed to assess apoptotic activity and astroglial responses in the cervical spinal cord. Caspase-3 immunostaining was used as a marker of apoptosis to determine whether diabetes-induced neuronal loss was associated with activation of programmed cell death pathways. In parallel, glial fibrillary acidic protein (GFAP) immunoreactivity was assessed to examine astrocyte activation and potential neuroinflammatory changes across experimental groups. These analyses aimed to provide complementary molecular and cellular insights into the neuroprotective effects of curcumin and to correlate immunohistochemical findings with quantitative stereological outcomes (Figures 5, 6).

## 4 Discussion

Curcumin, the principal polyphenolic compound extracted from the rhizomes of *Curcuma longa* (turmeric), has attracted considerable scientific attention due to its broad spectrum of pharmacological activities, including anti-inflammatory, antioxidant, anticancer, immunomodulatory, and neuroprotective properties (Alam et al., 2024; Azzini et al., 2024). These beneficial effects are primarily attributed to curcumin’s capacity to scavenge reactive oxygen species, activate the Nrf2/HO-1 antioxidant pathway, and suppress pro-inflammatory NF-κB signaling, thereby modulating key molecular mechanisms underlying a wide range of chronic and neurodegenerative disorders (He et al., 2021; Abdul-Rahman et al., 2024). Recent systematic reviews and meta-analyses have further confirmed curcumin’s anti-apoptotic and neuroprotective efficacy in experimental CNS injury models, demonstrating reductions in caspase-3 activity, increased Bcl-2 expression, and upregulation of neurotrophic factors (Azzini et al., 2024).

Chronic diabetic conditions are known to induce structural alterations in motor neurons, primarily through sustained hyperglycemia-associated metabolic stress and oxidative damage. These factors impair neuronal maintenance and growth mechanisms (Stefano et al., 2016; Wanrooy et al., 2018; Gonzalez et al., 2023). Experimental and clinical studies have demonstrated that diabetes leads to neuronal atrophy characterized by reduced soma size rather than immediate neuronal loss. This reflects impaired protein synthesis, mitochondrial dysfunction, and cytoskeletal alterations (Shimoshige et al., 2010; Kneynsberg et al., 2017). In parallel, insulin deficiency or insulin resistance in diabetes disrupts insulin/IGF-1 signaling pathways that are essential for neuronal survival and regulation of soma volume, thereby contributing to motor neuron shrinkage (Rethelyi et al., 2023).

Additionally, diabetes-associated reductions in neurotrophic support and microvascular dysfunction further exacerbate neuronal structural degeneration (Roman-Pintos et al., 2016; Hayden, 2019). Consistent with these mechanisms, our stereological results revealed a significant reduction in motor neuron soma size in the diabetic group. This finding confirms that diabetes promotes motor neuron atrophy as an early pathological feature of diabetic neuropathy and demonstrates the structural vulnerability of spinal motor neurons under chronic hyperglycemic conditions.

The increased ratio of white matter area to total spinal cord area observed in the DC3 group compared with the sham group may reflect diabetes-induced alterations in gray matter integrity rather than a true expansion of white matter. Diabetes is known to cause neuronal atrophy and reduction in neuronal soma size, particularly affecting motor neurons in the ventral horn, which leads to a relative decrease in gray matter volume (Sima et al., 1988). In addition, diabetes-associated neuroinflammation and glial activation can contribute to changes in tissue composition, including relative preservation or mild swelling of myelinated fiber tracts (Sima et al., 1983). Microvascular dysfunction and metabolic stress in diabetic conditions preferentially impair metabolically active neuronal cell bodies, while myelinated axons in white matter may be comparatively less affected at early or intermediate stages of diabetic neuropathy (Yang et al., 2025). Consequently, the apparent increase in the white matter–to–total area ratio in the DC3 group is likely a secondary effect of gray matter atrophy rather than an absolute increase in white matter volume.

Light microscopic examination revealed a chromatolytic response under diabetic conditions. As previously reported in the literature, this chromatolytic appearance arises from the redistribution and disorganization of Nissl bodies within the neuron following injury and represents a characteristic morphological hallmark of neuronal damage (Delibas and Kaplan, 2024). Chromatolysis is considered an adaptive cellular response that alters intracellular protein synthesis dynamics and overall neuronal activity, and similar findings have been consistently documented in earlier studies (Riancho et al., 2014; Moon, 2018). One limitation of the present study should be acknowledged. Although representative histological images were provided for most experimental groups, a corresponding light-microscopic image for the DC3 group was not available for the light-microscopic figure plate. While quantitative stereological analyses were performed systematically across all groups using identical sampling procedures, the absence of a representative micrograph for this group may limit the qualitative visual comparison of tissue morphology across treatment conditions. Another limitation of the study is that no quantitative immunohistochemical analysis was performed in comparison of the IHC staining. This limits the statistical interpretability of the IHC findings. Future studies should ensure complete histological documentation for all experimental groups to further strengthen the morphological interpretation of neuroprotective effects. We used only female rats to maintain consistency with our laboratory’s established protocols. While this approach ensures internal consistency and comparability with our previous works (Ulubay et al., 2020; Sahin et al., 2024; Tufekci et al., 2025), sex-specific differences in diabetic neuropathy and curcumin responsiveness have been reported in some studies (Sorge et al., 2015). Future research should include both male and female animals to assess potential sex differences in curcumin’s neuroprotective effects and to enhance the generalizability of findings.

The observation of comparable morphological features in the groups treated with curcumin after injury suggests that this chromatolysis response is not fully reversible under the applied experimental conditions. Furthermore, prominent vacuolization in the intercellular space of diabetic tissues reflects the severity of tissue damage and disruption of the extracellular microenvironment. Collectively, these findings indicate that diabetes impairs neuronal integrity and tissue morphology. In addition to stereological findings, the present immunohistochemical observations further support the cellular mechanisms underlying diabetic spinal cord pathology. Increased caspase-3 immunoreactivity in the diabetic group indicates activation of apoptotic pathways, consistent with the observed reduction in motor neuron number and soma size. Our observation of increased caspase-3 immunoreactivity in the diabetic spinal cord is consistent with prior evidence that experimental diabetes activates apoptotic signaling within spinal cord tissue, including increased cleaved caspase-3 in STZ models (Elsayed et al., 2023). The enhanced caspase-3 staining in specific treatment groups suggests that apoptosis-related processes may persist depending on the timing of curcumin administration. Furthermore, the increase in GFAP immunoreactivity, particularly in the DC2 group, reflects astrocyte activation and a reactive gliosis response, which is commonly associated with metabolic stress and neuroinflammation in diabetic conditions. The group-wise differences we observed in GFAP staining align with the literature indicating that diabetes disrupts astrocyte status in the spinal cord, with GFAP immunoreactivity showing model, region, and duration-dependent alterations (Afsari et al., 2008; Wang et al., 2025). Although these immunohistochemical evaluations were qualitative and based on morphological observation rather than quantitative analysis, the staining patterns provide supportive evidence that diabetes induces both apoptotic signaling and glial activation in the spinal cord. Together with the stereological data, these findings suggest that curcumin’s neuroprotective effects may be mediated, at least in part, through modulation of apoptosis and astroglial reactivity.

The present findings indicate that curcumin may partially protect spinal motor neurons from STZ-induced diabetic damage. The degree of neuroprotection was influenced by the timing of administration, with early or simultaneous treatment yielding better outcomes. Administration initiated at different stages of diabetic progression produced variable degrees of neuronal preservation, suggesting that curcumin may interact with disease mechanisms differently across pathological phases. Interestingly, the DC2 group, in which curcumin treatment was initiated later after diabetes induction, still showed a significant increase in motor neuron counts compared with untreated diabetic animals. This observation suggests that diabetic spinal cord pathology may retain a partially reversible therapeutic window even after structural alterations become evident, highlighting the potential of curcumin not only as a preventive but also as a therapeutic intervention. Simultaneous administration of curcumin with STZ exposure may reflect a prophylactic neuroprotective mechanism, possibly through attenuation of the early oxidative burst, suppression of inflammatory signaling cascades, and stabilization of mitochondrial function triggered by hyperglycemia. Such early modulation of pathogenic pathways may prevent downstream structural damage and neuronal loss. The DC3 group showed lower motor neuron counts than expected and performed worse than delayed treatment groups (DC1, DC2). This may be due to early curcumin interfering with adaptive stress responses, unfavorable metabolic effects during acute diabetes, and suboptimal timing of the 30 mg/kg dose. High variability in this group (CV = 33%) also suggests inconsistent responses. Overall, the findings indicate that curcumin is more effective when administered after diabetes is established rather than at onset. The absence of significant changes in total spinal cord volume suggests that diabetic pathology in our model primarily affects cellular composition rather than gross tissue architecture, which is consistent with the relatively short disease duration and indicates that volumetric changes may require longer progression.

Taken together, these stereological findings indicate that curcumin’s neuroprotective efficacy in diabetic spinal cord pathology depends critically on treatment timing, highlighting the importance of therapeutic windows in metabolic neurodegenerative disorders. Curcumin can provide partial neuroprotection against diabetes-induced motor neuron loss, with efficacy dependent on treatment timing. Although curcumin did not alter spinal cord volume, it attenuated motor neuron loss. However, the lack of significant protection in DC1 and DC3 groups indicates that curcumin’s neuroprotective effects may require optimization of dosing and timing. The mechanisms underlying this effect warrant further investigation.

## Data Availability

The original contributions presented in the study are included in the article/supplementary material, further inquiries can be directed to the corresponding author.
